# Uncommon presentation of a rare tumour - incidental finding in an asymptomatic patient: case report and comprehensive review of the literature on intrapericardial solitary fibrous tumours

**DOI:** 10.1186/s12885-017-3574-0

**Published:** 2017-09-02

**Authors:** Csilla Czimbalmos, Ibolya Csecs, Miklos Polos, Elektra Bartha, Nikolette Szucs, Attila Toth, Pal Maurovich-Horvat, David Becker, Zoltan Sapi, Zoltan Szabolcs, Bela Merkely, Hajnalka Vago

**Affiliations:** 1 Heart and Vascular Center, Semmelweis University, 68 Varosmajor St, Budapest, H-1122 Hungary; 2 2nd Department of Internal Medicine, Semmelweis University, Budapest, Hungary; 3 MTA-SE Cardiovascular Imaging Research Group, Semmelweis University, Budapest, Hungary; 4 1st Department of Pathology and Experimental Cancer Research, Semmelweis University, Budapest, Hungary

**Keywords:** Solitary fibrous tumour, Intrapericardial localization, Multimodality imaging, Long term follow-up, Case report

## Abstract

**Background:**

A solitary fibrous tumour is a rare, mainly benign spindle cell mesenchymal tumour most commonly originating from the pleura. An intrapericardial location of a solitary fibrous tumour is extremely unusual. We present a case of an asymptomatic patient with a slow-growing massive benign cardiac solitary fibrous tumour.

**Case presentation:**

A 37-year-old asymptomatic female patient was referred to our hospital with an enlarged cardiac silhouette found on her screening chest X-ray. The echocardiographic examination revealed pericardial effusion and an inhomogeneous mobile mass located in the pericardial sac around the left ventricle. Cardiac magnetic resonance (MRI) examination showed an intrapericardial, semilunar-shaped mass attached to the pulmonary trunk with an intermediate signal intensity on proton density-weighted images and high signal intensity on T2-weighted spectral fat saturation inversion recovery images. First-pass perfusion and early and late gadolinium-enhanced images showed a vascularized mass with septated, patchy, inhomogeneous late enhancement. Coronary computed tomography angiography revealed no invasion of the coronaries. Based on the retrospectively analysed screening chest X-rays, the mass had started to form at least 7 years earlier. Complete resection of the tumour with partial resection of the pulmonary trunk was performed. Histological evaluation of the septated, cystic mass revealed tumour cells forming an irregular patternless pattern; immunohistochemically, the cells tested positive for vimentin, CD34, CD99 and STAT6 but negative for keratin (AE1-AE3), CD31 and S100. Thus, the diagnosis of an intrapericardial solitary fibrous tumour was established. There has been no recurrence for 3 years based on the regular MRI follow-up.

**Conclusion:**

Intrapericardial SFTs, showing slow growth dynamics, can present with massive extent even in completely asymptomatic patients. MRI is exceedingly useful for characterizing intrapericardial masses, allowing precise surgical planning, and is reliable for long-term follow up.

**Electronic supplementary material:**

The online version of this article (10.1186/s12885-017-3574-0) contains supplementary material, which is available to authorized users.

## Background

A solitary fibrous tumour (SFT) is a rare primary tumour most commonly originating from mesenchymal tissue of the pleura. Complete surgical resection is the main treatment if possible. Histological appearance shows spindle-shaped cells and collagen fibres. Immunohistochemical features are vimentin, CD34, CD99 positivity and S-100 protein negativity [1]. Although the majority of SFTs generally exhibit clinically benign behaviour, 10–30% of SFTs have been associated with local recurrence or histologic malignancy [2, 3]. We have only limited data regarding the manifestation and behaviour of rare extrapleural forms such as cardiac SFT [4].

## Case presentation

**Fig. 1:**
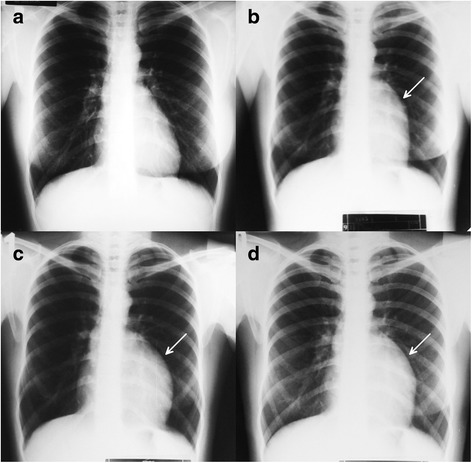
Chest X-ray examinations performed in 2004 (panel **a**), 2007 (panel **b**), 2010 (panel **c**) and in 2014 (panel **d**). *Arrows* show the enlarged cardiac silhouette

**Fig. 2:**
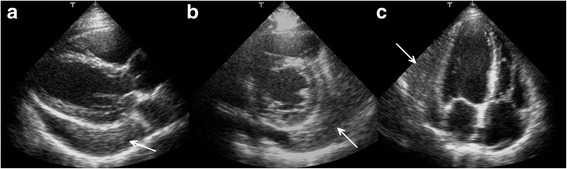
Transthoracic 2D echocardiography in the parasternal long-axis plane (panel **a**), short-axis plane (panel **b**) and apical four-chamber view (panel **c**). *Arrows* show the intrapericardial mass

**Fig. 3:**
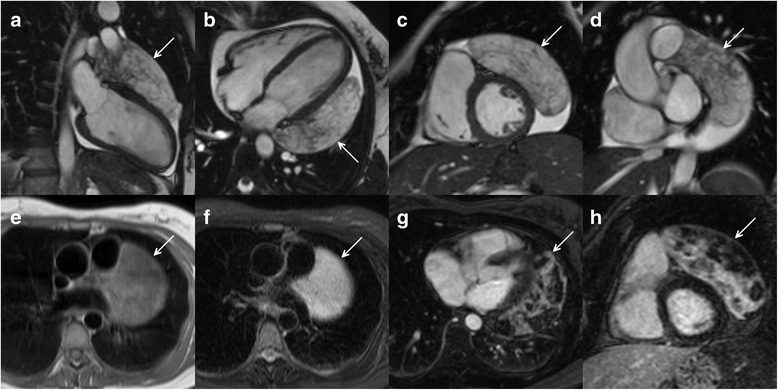
Cine movie MRI images in the long- (panel **a**, **b**) and short-axis planes in diastolic phase (panel **c** and **d**). Intermediate signal intensity on proton density-weighted images (panel **e**) and high signal intensity on T2-weighted SPIR images (panel **f**). LGE images in the long- (panel **g**) and short-axis planes (panel **h**). *Arrows* show the intrapericardial tumour

**Fig. 4:**
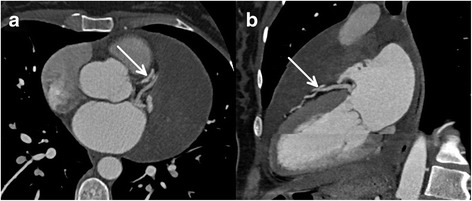
Coronary CTA images (panel **a**: axial plane, panel **b**: two-chamber view reconstruction) showed that coronary arteries were not invaded by the tumour. *Arrows* show the left anterior descending artery

**Fig. 5:**
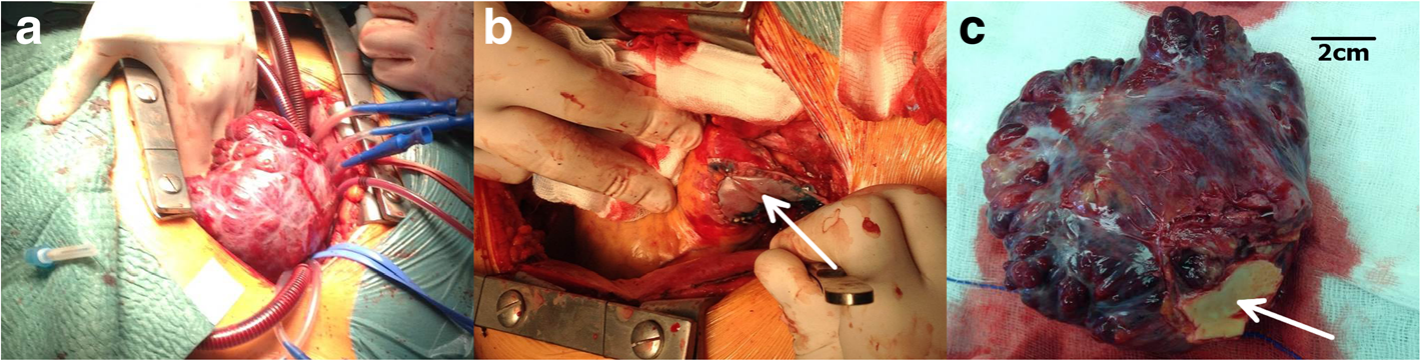
The intraoperative images show the complete resection of the tumour and partial resection of the pulmonary trunk (panel **a**). The pulmonary trunk was reconstructed using the pericardial patch technique (panel **b**). The encapsulated giant tumour with the size of 10 × 11 × 4 cm; the *arrow* shows the resected part of the pulmonary trunk (panel **c**)

**Fig. 6:**
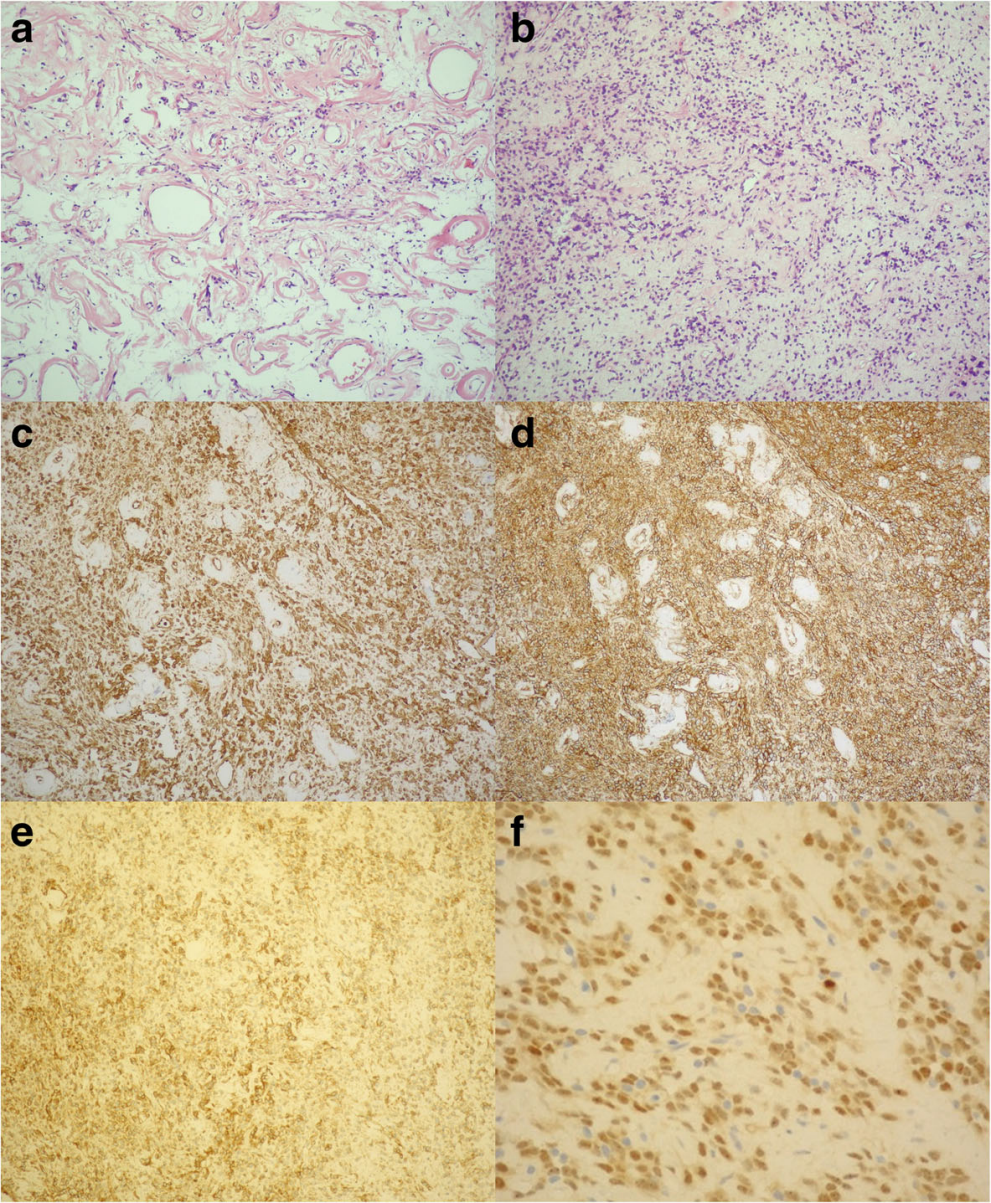
Histology: Haematoxylin and eosin, spindle-shaped cells with the “patternless pattern” (panel **a**, **b**). Immunohistochemistry: the cells were positive for vimentin (panel **c**), CD34 (panel **d**), CD99 (panel **e**) and STAT6 (panel **f**)

**Fig. 7:**
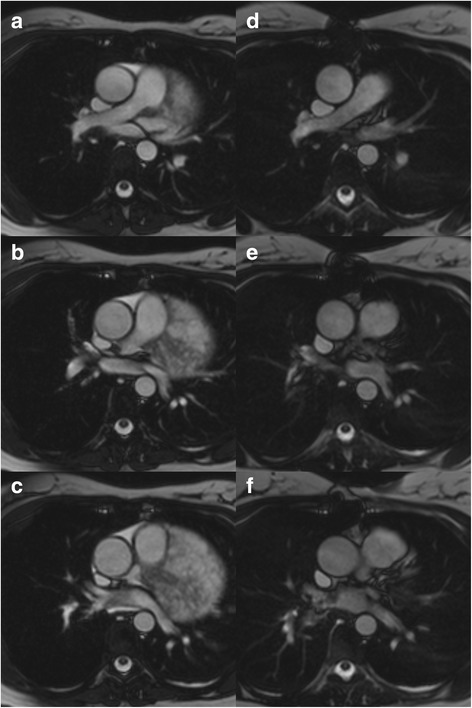
Cine movie MRI images in transverse planes in the diastolic phase before surgery (**a**-**c**) and at the three-year follow-up (**d**-**f**)



A 37-year-old asymptomatic female patient was referred to our hospital with an enlarged cardiac silhouette found on her screening chest X-ray (Fig. 1, panel d). She had no history of cardiovascular disease. She had a spontaneous abortion 3 years previously, and she is a mother of three children. She has a positive family history of cardiovascular diseases and cancer (lung adenocarcinoma and brain tumour). Physical examination revealed distant heart sounds, a regular rate and rhythm. The echocardiographic examination revealed pericardial effusion and an inhomogeneous mobile mass located in the pericardial sac around the left ventricle (Fig. 2, Additional file 1). Subsequently, cardiac magnetic resonance imaging (MRI) was performed, which showed an intrapericardial, semilunar-shaped mass with a size of 10 × 4 × 9 cm attached to the pulmonary trunk surrounding the aortic root, left atrium and left ventricle (Fig. 3). The tumour had well-demarcated margins and did not invade the blood vessels or myocardium. The caudal part of the tumour was mobile, while its cranial part was fixed to the pulmonary trunk (Additional file 2). The MRI scan detected intermediate signal intensity on proton density-weighted images and high signal intensity on T2-weighted spectral fat saturation inversion recovery (SPIR) images (Fig. 3, panel e, f). Both first-pass perfusion and early and late gadolinium-enhanced (LGE) images showed that the mass was vascularized and showed septated, patchy, inhomogeneous LGE (Fig. 3, panel g, h). Coronary computed tomography angiography (CTA) was performed to see whether the coronary arteries were affected. The coronary CTA proved that coronaries were not invaded by the tumour (Fig. 4). We have evaluated the previous screening chest X-rays of the patient that were acquired during the past 10 years. Based on these chest X-rays, we can conclude that the mass had started to form at least 7 years ago (Fig. 1). No abnormalities were found by the abdominal ultrasound examination, the results of hormone tests were normal, and a hormone-secreting nature of the tumour was excluded. Open heart surgery was indicated through median sternotomy (Fig. 5, panel a, b). The intraoperative findings confirmed the MRI and coronary CTA results. The tumour was intrapericardial, attached to the lateral wall of the pulmonary trunk, 2 cm distal from the commissures of the pulmonary valve. The tumour did not invade any other structures of the heart. Complete resection of the tumour with partial resection of the pulmonary trunk was performed (Fig. 5, panel c) using cardiopulmonary bypass. The pulmonary trunk was reconstructed with a round-shaped bovine pericardial patch (Fig. 5, panel b). The intra- and postoperative course were uneventful. Histological evaluation of the septated, cystic mass revealed tumour cells forming an irregular pattern, the so-called “patternless pattern” (Fig. 6, panel a, b). Immunohistochemically, the cells tested positive for vimentin, CD34, CD99 and STAT6 (Fig. 6, panel c-f) but negative for keratin (AE1-AE3), CD31 and S100. Thus, the diagnosis of a primary cardiac solitary fibrous tumour (SFT) was established. The tumour was classified as non-malignant because of the lack of increased mitotic activity, an intact capsule and no sign of vascular invasion. Regular and long-term clinical and MRI follow-up were indicated (every 6 months in the first year, later annually) because of the risk of late local recurrence. At the 3-year follow-up, the patient had no symptoms, and MRI did not show recurrence of the tumour (Fig. 7). The patient’s clinical history is summarized in a timeline, prepared in accordance with CARE guideline (Additional file 3).

**Additional file 1:** Transthoracic 2D echocardiography movie in the parasternal long-axis. (MP4 1.76 MB)




**Additional file 2:** Cine movie MRI images in the short-axis planes. (MP4 440 kb)


## Discussion and conclusions

A solitary fibrous tumour (SFT) is a rare spindle cell mesenchymal neoplasm that most commonly originates from the pleura, but for which extrapleural anatomic locations have also been reported (e.g., intraabdominal, meningeal, extracranial of the head and neck, and soft tissue SFT) [3, 5]. Primary cardiac SFTs are extremely rare; so far, there are only 11 cases reported in the English literature [6–16]. Additionally, primary pericardial SFT was mentioned in four publications without any specific information regarding the exact location, clinical data, patient symptoms or prognosis [17–20]. The case reports of primary intrapericardial SFTs reported in the literature are summarized in Table 1. Based on limited data about intrapericardial SFTs, it usually affects middle-aged patients, showing no gender-specific difference in incidence. Only two of them were diagnosed as malignant; in two cases, no information was available about the malignancy. Other than two asymptomatic patients with an incidental diagnosis, all of the reported patients with primary cardiac SFTs were symptomatic. Symptoms may depend on the extent and anatomic location of the tumour, most commonly dyspnoea, fatigue, chest discomfort/distress, palpitation, syncope or peripheral oedema.

**Table 1:** Case reports of primary intrapericardial SFTs reported in the literature supplemented with our case report

	Authors	Year	Age	Sex	Symptoms	Origin	Tumour imaging	Malignancy	Follow-up length (imaging modality)	Immuno-histochemical marker
1	Bortolotti U et al. [6]	1992	60	M	fatigue, chest discomfort, dyspnoea	AAo, PT	X-ray, echo, CT	Benign	9 m (X-ray, echo)	vimentin+
2	Seqawa D et al. [7]	1995	50	F	dyspnoea, palpitation	RV	X-ray, CT, MRI	NA	19 m (NA)	vimentin+
3	Flemming P et al. [8]	1996	53	F	NA	LV	NA	NA	died port HTX	CD34 + vimentin +
4	Andreani SM et al. [9]	1998	60	M	exertional dyspnoea	NA	X-ray, CT	Benign	4 y (NA)	NA
5	Corgnati G et al. [10]	2004	30	M	peripheral oedema	AAo, PT	X-ray, echo CT, MRI	Benign	18 m (NA)	NA
6	Bothe W et al. [11]	2005	39	F	palpitation	RA	echo	Benign	12 m (echo)	CD34 + vimentin +
7	Croti UA et al. [12]	2008	5 m	M	asymptomatic	LA	X-ray, echo	Benign	non-CV death after 6 m (NA)	CD34 +
8	Zhao XG et al. [13]	2012	55	M	chest distress, dyspnoea	RA	X-ray, CT	Malignant	died after surgery	CD34 +
9	Taguchi S et al. [14]	2013	49	F	asymptomatic	LV	CT, MRI	Malignant	NA	CD34 + vimentin + CD99 +
10	Bianchi G et al. [15]	2013	68	F	dyspnoea, fatigue	LV	echo, CT, MRI	Benign	12 m (echo)	CD34 + vimentin + Bcl2 +
11	Tamenishi A et al. [16]	2013	30	F	syncope	left PA	X-ray, CT	Benign	6y (NA)	CD34 +
12	Our case	2017	37	F	asymptomatic	PT	X-ray, echo, CT, MRI	Benign	3y (MRI)	CD34 + vimentin + CD99 + STAT6 +

*M* male, *F* female, *NA* no data available, *AAo* Ascending aorta, *PT* pulmonary trunk, *RA* right atrium, *LA* left atrium, *LV* left ventricle, *PA* pulmonary artery, *m* months, *y* years

As a first-line imaging modality, chest X-ray typically shows marked cardiomegaly, and echocardiography could verify an intrapericardial mass and pericardial effusion. MRI can provide additional information about the morphology, location and extent of intrapericardial masses, and it can help to further characterize the tumour tissue. In general, MRI characteristics of SFT vary because of the altering degree of vascularity, cystic degeneration, haemorrhage and necrosis. SFTs usually show intermediate signal intensity on T1-weighted images and variable signal intensity on T2-weighted images [2]. In our case, the detailed assessment of the tumour using various MRI sequences and contrast administration showed specific characteristics of extrapleural SFTs including septated, patchy, inhomogeneous LGE. Coronary CT angiography also has an added value in the precise evaluation of the relation with the coronary arteries.

SFT shows characteristic CD34 expression in 95% of the cases, and CD99 can also be positive [5]. However, these markers are not specific. According to recent studies, STAT6 is a highly sensitive and specific immunohistochemical marker of SFT [21]. Most of the intrapericardial SFTs confirmed vimentin and CD34 positivity; specific STAT6 immunostaining was not yet revealed in intrapericardial SFTs.

Our case confirms the conjecture that intrapericardial SFTs are typically slow-growing masses because, according to the consecutive X-ray images, the intrapericardial mass started to form at least 7 years ago.

Although the majority of SFTs of the thorax are benign and are cured by complete resection, 10–20% are locally aggressive or malignant [22]. Malignant histology is strongly associated with recurrence, but some benign SFTs still behave aggressively. The literature data imply a higher risk for the recurrence of extrapleural than that for pleuropulmonary SFT [23]; many recurrent SFTs do not respond to treatment. This underscores the need for continued long-term follow-up using a high-resolution, non-invasive imaging technique. In the reported cases, X-ray or echocardiography was used during follow-up, and only two intrapericardial SFT cases were reported with a follow-up longer than 2 years [9, 16]. An international registry would be needed to have more detailed information based on long-term follow-up regarding the recurrence tendency of intrapericardial SFTs.

Although intrapericardial SFT is an extremely rare condition, the slow-growth, considerable size of the tumour, and its typical MRI appearance can raise the suspicion of SFT. The “patternless pattern” histopathological finding, vimentin, CD34 and CD99 positivity and specific STAT6 immunostaining can be valuable indicators of this rare mesenchymal tumour. Owing to the high recurrence rate of extrapleural SFTs, long-term follow-up is crucial, and magnetic resonance imaging is a reliable method for the early detection of local recurrence.

## Additional files

Additional file 3: The patient’s clinical history organized as a timeline. (PDF 287 kb)

## Abbreviations

MRI: Magnetic resonance imaging
SFT: Solitary fibrous tumour

## References

[1] Geramizadeh B, Marzban M, Churg A (2016). Role of immunohistochemistry in the diagnosis of solitary fibrous tumor, a review. Iran J Pathol.

[2] Sung SH, Chang JW, Kim J, Lee KS, Han J, Park SI (2005). Solitary fibrous tumors of the pleura: surgical outcome and clinical course. Ann Thorac Surg.

[3] Demicco EG, Park MS, Araujo DM, Fox PS, Bassett RL, Pollock RE (2012). Solitary fibrous tumor: a clinicopathological study of 110 cases and proposed risk assessment model. Mod Pathol.

[4] Taguchi S (2015). Primary Cardiac Solitary Fibrous Tumors. Ann Thorac Cardiovasc Surg.

[5] Hasegawa T, Matsuno Y, Shimoda T, Hasegawa F, Sano T, Hirohashi S (1999). Extrathoracic solitary fibrous tumors: their histological variability and potentially aggressive behavior. Hum Pathol.

[6] Bortolotti U, Calabro F, Loy M, Fasoli G, Altavilla G, Marchese D (1992). Giant intrapericardial solitary fibrous tumor. Ann Thorac Surg.

[7] Segawa D, Yoshizu H, Haga Y, Hatori N, Tanaka S, Aida S (1995). Successful operation for solitary fibrous tumor of the epicardium. J Thorac Cardiovasc Surg.

[8] Flemming P, Maschek H, Werner M, Kreft A, Graeter T, Georgii A (1996). Solitary fibrous tumor of the epicardium. Pathologe.

[9] Andreani SM, Tavecchio L, Giardini R, Bedini AV (1998). Extrapericardial solitary fibrous tumour of the pericardium. Eur J Cardiothorac Surg.

[10] Corgnati G, Drago S, Bonamini R, Trevi GP, Carra R, Di Summa M (2004). Solitary fibrous tumor of the pericardium presenting itself as a pericardial effusion and right ventricular obstruction. J Cardiovasc Surg.

[11] Bothe W, Goebel H, Kunze M, Beyersdorf F (2005). Right atrial solitary fibrous tumor - a new cardiac neoplasm?. Interact Cardiovasc Thorac Surg.

[12] Croti UA, Braile DM, Moscardini AC, Cury PM (2008). Solitary fibrous tumor in a child’s heart. Rev Bras Cir Cardiovasc.

[13] Zhao XG, Wang H, Wang YL, Chen G, Jiang GN (2012). Malignant solitary fibrous tumor of the right atrium. Am J Med Sci.

[14] Taguchi S, Mory A, Yamabe K, Suzuki R, Nishizawa K, Hasegawa I (2013). Malignant Solitary Fibrous Tumor of the Left Ventricular Epicardium. Ann Thorac Surg.

[15] Bianchi G, Ferrarini M, Matteucci M (2013). Giant solitary fibrous tumor of the epicardium causing reversible heart failure. Ann Thorac Surg.

[16] Tamenishi A, Matsumura Y, Okamoto H (2013). Solitary fibrous tumor causing cardiac tamponade. Ann Thorac Surg.

[17] Roggli VL, Kolbeck J, Sanfiliippo F, Shelburne JD, Rosen PP, Fecher RE (1987). Pathology of human mesothelioma. Etiologic and diagnostic considerations. Pathology annual.

[18] El-Naggar AK, Ro JY, Ayala AG, Ward R, Ordonez NG (1989). Localized fibrous tumor of the serosal cavities. Immunohistochemical, electron-microscopic, and flow-cytometric DNA study. Am J Clin Pathol.

[19] Odim J, Reehal V, Laks H, Mehta U, Fishbein MC (2003). Surgical pathology of cardiac tumors. Two decades at an urban institution. Cardiovasc Pathol.

[20] Burke A, Virmani R, Burke A, Virmani R (1996). Classification and incidence of cardiac tumors. Tumors of the heart and great vessels. Atlas of tumor pathology.

[21] Doyle LA, Vivero M, Fletcher CD, Mertens F, Hornick JL (2014). Nuclear expression of STAT6 distinguishes solitary fibrous tumour from histologic mimics. Mod Pathol.

[22] Chick JF, Chauhan NR, Madan R (2013). Solitary fibrous tumours of the thorax: nomenclature, epidemiology, radiologic and pathologic findings, differential diagnoses, and management. AJR Am J Roentgenol.

[23] Wilky BA, Montgomery EA, Guzzetta AA, Ahuja N, Meyer CF (2013). Extrathoracic location and "borderline" histology are associated with recurrence of solitary fibrous tumors after surgical resection. Ann Surg Oncol.

