# Supplementary material for: Uncommon presentation of a rare tumour - incidental finding in an asymptomatic patient: case report and comprehensive review of the literature on intrapericardial solitary fibrous tumours
Source: BMC Cancer. 2017 Sep 2;17:612. doi: 10.1186/s12885-017-3574-0 (PMC5581469; doi:10.1186/s12885-017-3574-0)
Supplement: Supplementary file 3 — The patient’s clinical history organized as a timeline. (PDF 287 kb) [file 12885_2017_3574_MOESM3_ESM.pdf]

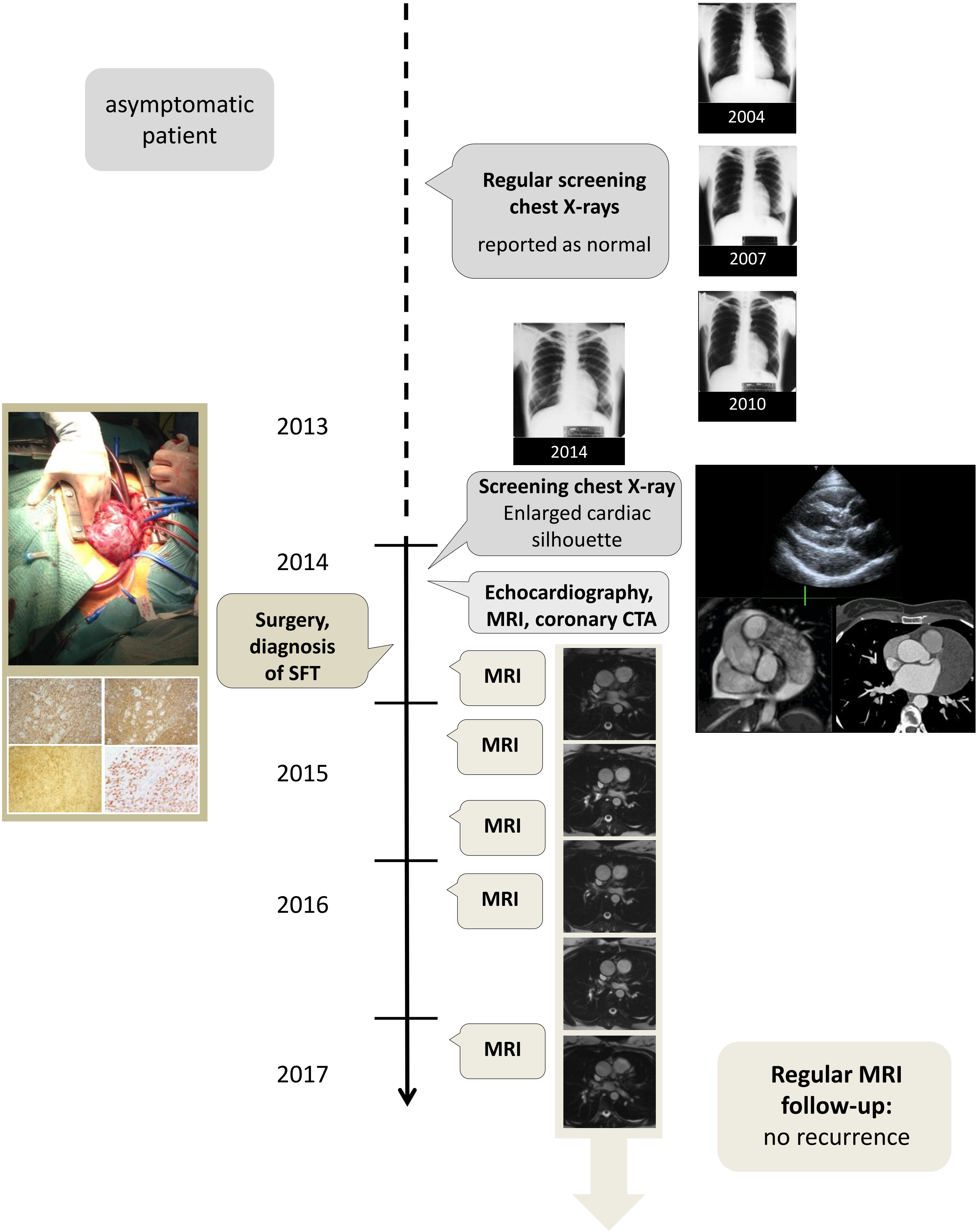

asymptomatic patient
Regular screening chest X-rays
reported as normal
2004
2007
2010
2013
2014
Screening chest X-ray
Enlarged cardiac silhouette
Echocardiography, MRI, coronary CTA
Surgery, diagnosis of SFT
MRI
MRI
2015
MRI
2016
MRI
MRI
2017
Regular MRI follow-up:
no recurrence
